# School-based support for emotion-related attendance challenges: effectiveness of @School when implemented with neurodiverse adolescents, their parents, and school staff

**DOI:** 10.3389/fpsyg.2025.1613712

**Published:** 2025-06-23

**Authors:** Evelyne Karel, Cynthia Defourny, Gil Keppens, Patricia A. Graczyk, Floor Sauter, David Heyne

**Affiliations:** ^1^Department of Education and Innovation, De Berkenschutse, Centre for Educational Expertise, Heeze, Netherlands; ^2^Department of Sociology, Tilburg University, Tilburg, Netherlands; ^3^Department of Psychiatry, University of Illinois at Chicago, Chicago, IL, United States; ^4^De Banjaard, Outpatient Mental Healthcare Expertise Center for Youth with MID/BIF (Youz, Parnassia Group Psychiatric Institute), The Hague, Netherlands; ^5^Institute of Psychology, Leiden University, Leiden, Netherlands; ^6^School of Psychology, Faculty of Health, Deakin University, Burwood, VIC, Australia

**Keywords:** emotion-related school attendance challenges, school refusal, neurodiversity, school-based intervention, cognitive behavioral therapy, school attendance, anxiety, depression

## Abstract

Emotion-related school attendance challenges (ER-SAC) among neurodiverse adolescents are a growing concern due to their impact on both academic and social–emotional development. Despite the prevalence of ER-SAC, few school-based interventions specifically addressing this challenge have been evaluated in real-world settings. The current effectiveness study examined outcomes of the @School intervention, a modular, developmentally sensitive cognitive behavioral intervention that had previously only been evaluated in a research context. In this study, it was delivered by school-based psychologists in a specialized educational setting. Nineteen neurodiverse adolescents aged 12–17 years, all experiencing ER-SAC, participated in the study along with their parents. @School comprises individualized modules for adolescents and parents, as well as structured collaboration with school staff. Outcomes were assessed at pre-, post-, and five-month follow-up, and included adolescents’ school attendance, anxiety, school-related fear, depression, and self-efficacy, together with parent self-efficacy. Results revealed significant improvements in school attendance, adolescent anxiety (reported by both adolescents and parents), adolescent depression (reported by parents), and school-related fear. No significant changes were observed in adolescent or parent self-efficacy. Post-hoc analyses indicated significant reductions in social anxiety symptoms, although these reductions did not predict school attendance outcomes. Findings support the effectiveness of the @School intervention, delivered in a real-world setting, for improving school attendance and reducing emotional distress among neurodiverse adolescents. The results also highlight the value of school-based interventions that integrate support across multiple levels—addressing the needs of adolescents, their parents, and the school environment—to respond to the complex emotional and contextual factors contributing to ER-SAC.

## 1 Introduction

School attendance challenges (SAC) are a significant and growing concern worldwide. They are particularly prevalent among neurodiverse adolescents (Adams et al., 2019; Adams et al., 2025; Munkhaugen et al., 2017; Sasso and Sansour, 2024), defined in this study as those diagnosed with autism spectrum disorder (ASD) and/or attention deficit hyperactivity disorder (ADHD). Furthermore, SACs are associated with long-term consequences for academic achievement and mental health (Heyne et al., 2022a; Kearney et al., 2022) Within the broad array of SACs, Emotion-Related School Attendance Challenges (ER-SAC) represent a particularly complex form driven by emotional distress (Heyne et al., 2019). Although ‘school refusal’ was a commonly used term, consensus on terminology is evolving, and we use ER-SAC to reflect the shift toward more student- and parent-centered language. Given the potential impact of ER-SAC on adolescent development, there is an urgent need for interventions that not only support school attendance but also address the emotional factors contributing to absence.

Adolescence is a critical developmental period for ER-SAC, marked by increasing complexity in social, academic, and psychological demands, which can complicate intervention efforts (Heyne, 2022). Adolescents experiencing ER-SAC often demonstrate a poorer response to intervention compared to children, a pattern linked to the increasing complexity of social, academic, and psychological challenges in adolescence (Heyne, 2022). The transition to secondary education presents heightened academic demands and greater autonomy expectations, which can exacerbate SACs (Melvin et al., 2025). These developmental challenges underscore the importance of tailoring interventions to the unique psychological, academic, and social needs that emerge during adolescence (Waite and Creswell, 2014).

Adolescent mental health difficulties are among the most significant influences on school attendance, representing a growing global concern due to rising prevalence rates (World Health Organization, 2021). Currently, one in seven (14%) individuals aged 10–19 years experiences a mental disorder, accounting for 15% of the global disease burden in this age group (World Health Organization, 2021). Anxiety and depressive disorders, in particular, are common and have significant implications for school attendance and academic performance (Finning et al., 2019; Finning et al., 2022; World Health Organization, 2021). These conditions often contribute to social withdrawal, isolation, and loneliness, further exacerbating the risk of school absence (World Health Organization, 2021). Because anxiety and depression are key early drivers of school non-attendance (Adams et al., 2025; Heyne et al., 2019), there is a need to integrate emotional support into interventions for ER-SAC.

Neurodiverse adolescents are Particularly vulnarable to factors contributing to ER-SAC (Fleming et al., 2017, 2020; Nordin et al., 2023; World Health Organization, 2021). Increasingly, research suggests that adolescents facing neurodiversity have a heightened risk of school non-attendance due to, among other factors, increased susceptibility to mental health difficulties, particularly anxiety and depression (Adams, 2021; Morales-Hidalgo et al., 2023; Munkhaugen et al., 2017; Orm et al., 2024; Totsika et al., 2020). Recent findings by Adams et al. (2025) suggest that, among children with autism, anxiety is more strongly linked to school non-attendance than any other factor examined. Additional factors contributing to school non-attendance among neurodiverse adolescents include an increased likelihood of experiencing bullying (Bitsika et al., 2020; McClemont et al., 2020; Ochi et al., 2020), familial influences (Niemi et al., 2022), and insufficient accommodations within current educational settings (Connolly et al., 2023; Li et al., 2024; May et al., 2021; White et al., 2024).

Research also shows that neurodiverse adolescents have a higher likelihood of school non-attendance compared to their neurotypical peers (Adams, 2021; Black and Zablotsky, 2018; Niemi et al., 2022; Totsika et al., 2020). ER-SAC is a particularly prevalent type of attendance challenge among neurodiverse adolescents, with rates substantially higher than the 1–7% observed in the general population. Estimates of school non-attendance in adolescents with autism spectrum disorder (ASD) range from 9% to as high as 72.6% (Adams, 2021; Totsika et al., 2020), while in adolescents with ADHD, reported rates vary between 5.2 and 16%, depending on age and context (Black and Zablotsky, 2018; Niemi et al., 2022). Comorbid anxiety has been shown to further increase the likelihood of school non-attendance in these groups (Niemi et al., 2022). Totsika et al. (2020) found that ER-SAC accounted for 43% of non-attendance in their study population of neurodiverse adolescents. Further, Adams (2021) highlighted that ER-SAC contributed to half of all recorded full-day and partial-day school absences, emphasizing its role as a primary factor in chronic absence.

Cognitive Behavioral Therapy (CBT) is a widely used intervention for ER-SAC, among both neurotypical and neurodiverse adolescents, (Finning et al., 2022; Heyne et al., 2020; Johnsen et al., 2021; Maynard et al., 2018), yet it fails to adequately support one-third to two-thirds of adolescents displaying ER-SAC (Heyne, 2023). The Maynard et al. (2018) review found that CBT significantly improved school attendance but did not significantly reduce anxiety symptoms. Improved school attendance is a valuable outcome, as it allows adolescents to access academic, social, and emotional resources that support their development. However, increasing time at school alone is not sufficient. Emotional support is essential to disrupt patterns of avoidance and promote sustained school engagement and psychological well-being (Heyne et al., 2022b; Finning et al., 2019). To achieve both improved attendance and reduced emotional distress, interventions must adopt a multi-level approach that addresses the psychological and contextual factors contributing to ER-SAC (Melvin et al., 2019; Sasso and Sansour, 2024).

The @School intervention (Heyne and Sauter, 2013) is a developmentally sensitive and modular CBT-based program designed to support adolescents with ER-SAC through a multi-system approach. It addresses individual, family, and school systems—enabling a comprehensive response to the complex interplay of factors that contribute to ER-SAC. It includes 12 youth-focused and 12 parent-focused modules, composed of both standard and optional modules that allow for individual tailoring based on a carefully prepared case formulation. The school-focused modules foster school staff involvement in addressing academic, emotional, behavioral, organizational, and social factors that may impact attendance. Examples of adolescent modules include: ‘Solving Problems’, ‘Managing Stress’, and ‘Attending School’, while parent modules include ‘Thinking about the Teenage Years’, ‘Addressing Maintenance Factors’, and ‘Facilitating School Attendance’. Simultaneously, the intervention engages schools to create supportive environments for academic and socio-emotional growth, ensuring that systemic factors contributing to ER-SAC are addressed. Recognizing that sustainable support for school attendance requires alignment across the individual, family, and school systems, the @School intervention integrates them into a cohesive approach, through coordinated goal setting, joint sessions, and shared communication strategies involving adolescents, parents, and school staff. A complete overview of all modules, including their objectives and key techniques, is provided in Supplementary material 1. For additional background and theoretical rationale, a more detailed description of the intervention is available in Heyne and Sauter (2013), and interested readers may contact the first author for access to the manual.

The Heyne et al. (2011) efficacy study provided support for the @School intervention under controlled research conditions. These conditions included strict inclusion and exclusion criteria (e.g., no adolescents with autism), standardized intervention delivery in a clinical setting, and therapist adherence monitoring to ensure fidelity to the treatment protocol. Study results showed significant improvements in school attendance, reductions in school-related anxiety, and enhanced self-efficacy among adolescents and parents. These findings point to the potential of a developmentally sensitive modular CBT approach—one that is gounded in a multi-level framework—to meaningfully support adolescents experiencing ER-SAC and their families.

The @School intervention was initially evaluated under standardized research conditions. Since then, related initiatives have emerged that build on its principles. One such initiative is the Back2School (B2S) program, which adapts core elements of @School for broader application (Johnsen et al., 2024; Thastum et al., 2019). The current study contributes to this body of work by evaluating the original @School intervention in a real-world educational context, specifically a school for students with special educational needs. Data collection for the study began in 2017 and proceeded slowly due to the modest resources available for the project, limited capacity in terms of available therapeutic staff, and disruptions caused by the COVID-19 pandemic.

The question remains as to whether the positive outcomes reported in the 2011 efficacy study of the @School intervention, conducted under optimal research conditions, extend to implementation in a real-world setting and with neurodiverse adolescents experiencing ER-SAC. We contribute to the literature by examining the effectiveness of the @School intervention in an educational context, specifically targeting neurodiverse adolescents with ER-SAC. In general, research on ER-SAC interventions has largely been conducted in controlled settings (Hannan et al., 2019; Heyne et al., 2011; Heyne, 2023; Tolin et al., 2009). Effectiveness studies, which assess interventions in real-world settings, are crucial for understanding how interventions function outside controlled conditions. They account for the complexities of school environments, the practical challenges of implementation, and the diversity of youth receiving the intervention, many of whom would be excluded from controlled trials due to comorbidity. The current study offers insight into the functioning of an existing intervention when delivered by school-affiliated psychologists in a real-world educational context.

This study was developed in response to a gap observed in practice and noted in the literature. That is, while professionals in clinical settings often prioritize the emotional well-being of adolescents displaying ER-SAC, they may not explicitly address school reintegration (Heyne and Sauter, 2013). Conversely, professionals in education settings may prioritize school attendance while overlooking underlying emotional barriers, potentially limiting sustained progress for adolescents (Thambirajah et al., 2008). This study assessed the effectiveness of the @School intervention, embedded within a school setting and addressing mental health needs, for neurodiverse adolescents experiencing ER-SAC. This also permits comparison with outcomes from the original efficacy study (Heyne et al., 2011), evaluating whether similar benefits are found when the intervention is delivered under real-world conditions. To evaluate the impact of this intervention, a non-randomized trial has been conducted within a special educational needs school, delivered by school-based psychologists. We hypothesized that the intervention would lead to increased school attendance and reductions in school-related fear and anxiety. Additionally, we expected decreases in depressive symptoms and improvements in adolescent and parent self-efficacy.

## 2 Method

### 2.1 Participants

#### 2.1.1 Study context

Adolescents in the study were enrolled at De Berkenschutse, an expertise center for specialized education, located in the south of the Netherlands. This school provides education and training for adolescents with epilepsy, long-term illness, learning difficulties, multiple disabilities, and neurodevelopmental problems.

#### 2.1.2 Criteria for inclusion and exclusion

Adolescents aged 10–17 years were included in the study if they met the criteria for ER-SAC presented in Heyne et al. (2011) and refined for the current study: (a) school attendance was below 80% in the prior 2 weeks, (b) parents confirmed awareness of their child’s whereabouts during each absence in the 2 week period; (c) the adolescent’s level of anxiety and/or depression was above the clinical cutoff based on adolescent or parent reports of emotional distress, assessed via standardized instruments (see Section 2.3.2); (d) the adolescent displayed no severe externalizing behavioral problems; (e) both the adolescent and parents demonstrated willingness to support the adolescent’s school attendance; and (f) the adolescent did not meet criteria for truancy.

Adolescents were excluded according to the following criteria: (a) IQ score below 80; (b) no medical clearance by the primary physician for treatment initiation; (c) presence of a DSM-5 psychotic disorder (American Psychiatric Association, 2022); (d) significant communication challenges or selective mutism; (e) developing personality disorder; (f) presence of addiction issues; (g) presence of eating disorders; (h) presence of severe suicidality; (i) other ongoing intensive treatment; (j) Post Traumatic Stress Disorder (k) changes in pharmacological treatment during the intervention period; and (l) severe conflict arising from a hostile parental divorce.

Eligibility for inclusion was determined through a two-stage process: an initial telephone screening conducted with one of the therapists of the @School intervention (Gate 1), during which preliminary information was collected to assess the likelihood of meeting the inclusion criteria, followed by a comprehensive pre-intervention assessment conducted with one of the therapists of the @School intervention (Gate 2), incorporating clinical interviews and standardized measures (see Section 2.2.2).

Sixty-six families were referred for telephone screening, with 33 families meeting the preliminary criteria and participating in pre-intervention assessment. Following assessment, 7 families (21%) were excluded due to: absence of clinical anxiety or depression (*n* = 1), severe externalizing behavioral problems (*n* = 1), psychiatric issues requiring (inpatient) care (*n* = 3), and transition to another school (*n* = 2).

#### 2.1.3 Description of included participants

Of the 26 families initially enrolled in the study, 3 withdrew during the intervention due to either a lack of motivation because the adolescent achieved full school attendance (*n* = 1), or a preference not to have to attend scheduled appointments (*n* = 2). A decision was made to exclude 4 of the remaining 23 families because they received more than 25% of their treatment online during COVID-19 lockdowns. Their experience of the intervention differed substantially from families who participated under pre-pandemic or post-pandemic conditions. The remaining 19 families completed post-intervention assessments, with 17 participating in follow-up assessments.

The completer sample comprised 10 females and 9 males, aged 12–17 years (M = 14, SD = 1.3). Diagnoses included ASD (*n* = 15), ADHD (*n* = 2), and other neurodevelopmental disorders (*n* = 2). The majority (79% *n* = 15) pursued higher general or pre-university secondary education, with adolescents distributed across the first to fourth years of secondary education in the Dutch school system. Family composition included 84% two-parent households (*n* = 16) and 16% single-parent households (*n* = 3).

Using the internationally recognized Tier system (Kearney, 2016; Kearney and Graczyk, 2020), all adolescents (100%) were classified as chronically absent (≤90% attendance, Tier 3) before the intervention.

### 2.2 Procedure

#### 2.2.1 Ethics

During pre-intervention assessment, adolescents and parents were briefed about the study and asked to provide written consent for participation. For ethical reasons, families were not denied intervention if they chose not to participate in the study, although this scenario did not occur in practice. Because the study evaluated standard practices at De Berkenschutse, it received a waiver from the ethics committee at Leiden University Medical Centre.

#### 2.2.2 Pre-intervention assessment

Families were referred to the LANS program, a Dutch acronym for ‘Leerlingen Allemaal Naar School’ (‘All Students to School’), a school-based initiative that includes the @School intervention as a core component. Referrals were made either by team leaders in the school’s internal care team or by the school’s admissions team for newly enrolled adolescents.

These teams identified adolescents displaying ER-SAC who and were considered likely to benefit from the intervention provided by LANS.

Families referred to the @School intervention by the internal care team or the admissions department participated in telephone screening conducted by one of the @School intervention therapists. If screening indicated that the inclusion criteria were likely to be met, families proceeded to a pre-intervention assessment, conducted in two in-person sessions at the school. These sessions, spaced 1 week apart, involved a semi-structured clinical interview with the adolescent and their parents, conducted by separate therapists. Families also completed questionnaires at home (see Section 2.3).

#### 2.2.3 Intervention

The @School intervention (Heyne and Sauter, 2013) incorporates 12 youth-focused and 12 parent-focused modules. As described in the manual, individualized intervention per family was based on a case formulation developed using information from the pre-intervention assessment. Individualization included the selection, dosing, and tailoring of modules. A flexible reintegration approach was applied, adjusting the pace of physical school attendance to the individual capacity of the adolescent. In some cases, school attendance was gradually increased from the start of the intervention, while in others, initial treatment was prioritized before systematically building up school participation. Sessions were conducted weekly, with individual sessions lasting 45 min and family sessions (where the adolescent, parents, and therapists participated) lasting 90 min. On average, adolescents attended 15 individual intervention sessions (*M* = 15.22, *SD* = 4.01), while parents attended 11 individual sessions (*M* = 10.84, *SD* = 2.83). In addition, families participated in 2 to 3 joint sessions (*M* = 2.5, *SD* = 0.86) involving both the adolescent and their parents, focusing on family communication and problem solving. Booster sessions were optional for all families, only five adolescents opted to participate. Collaboration with school staff was actively integrated through multidisciplinary meetings approximately every 2 weeks, involving therapists, school staff, and parents—and, when possible, the adolescent as well. These meetings served to align academic, emotional, and behavioral support strategies and to coordinate shared planning for school reintegration.

#### 2.2.4 Post-intervention and follow-up assessments

Each family participated in a post-intervention assessment scheduled 2 weeks after the intervention ended, and a two-month follow-up assessment thereafter. On average, post-intervention assessments were conducted approximately 2.4 weeks after the completion of the intervention, while follow-up assessments were conducted on average 5.4 months after intervention completion. Although follow-up was planned at 3 months post-intervention, variation in questionnaire return times led to a longer average follow-up period. All questionnaires were completed at home.

#### 2.2.5 Therapists and supervision

Four psychologists conducted the telephone screening, pre-intervention assessment, and intervention sessions. Two of the psychologists had postgraduate degrees and registrations in health care psychology, one had a postgraduate degree in school psychology, and one was completing the final year of postgraduate education in health care psychology. Three of the four had participated in a 100-h post-masters training in CBT. To support treatment fidelity, monthly group supervision sessions were held, totaling approximately 30 h and guided by one of the co-authors of the @School intervention. Supervision encompassed discussions on case formulations and treatment sessions, ensuring adherence to the intervention manual while optimizing therapeutic outcomes.

### 2.3 Measures

Outcomes were the adolescents’ school attendance and emotional distress (symptoms of anxiety, symptoms of depression, and school-related fear) as well as adolescents’ and parents’ self-efficacy.

#### 2.3.1 School attendance

The percentage of school attendance over the 20 school days preceding the assessment (i.e., pre-, post-, or follow-up assessment) was based on: (a) school records, in which teachers at the school had recorded attendance for every hour of the school day; or (b) attendance records from the previous school, for adolescents whose pre-intervention assessment occurred shortly after their enrolment at the school. Attendance status was categorized based on school attendance percentages, with Tier 1 representing good attendance (>95%), Tier 2 moderate attendance (90–95%), and Tier 3 chronic absence (≤90%). These classifications were used to analyze attendance outcomes over time.

#### 2.3.2 Emotional distress

Anxiety symptoms were measured using two validated instruments. First, adolescents and parents completed the Multidimensional Anxiety Scale for Children (MASC/MASC-P; March et al., 1997), Dutch version by Utens and Ferdinand (2000), the instrument used in the original 2011 study. The MASC and MASC-P contain 39 items rated on a 4-point Likert scale and assess four anxiety domains: physical symptoms, social anxiety, avoidance of harm, and separation anxiety. The MASC has been widely recognized as a reliable measure for evaluating treatment outcomes (Brooks and Kutcher, 2003) and continues to be applied in research on youth anxiety (Franke et al., 2024). The total score ranges between 0 and 117, with higher scores indicating greater anxiety severity.

Second, adolescents completed the Screen for Child Anxiety Related Emotional Disorders (SCARED; Monga et al., 2000), Dutch version by Muris et al. (2007), an instrument routinely used at the school where the study was conducted. The SCARED consists of 69 self-report items measuring symptoms across multiple anxiety disorders, including separation anxiety, panic disorder, specific phobia, social phobia, obsessive-compulsive disorder, post-traumatic and acute stress disorder, and generalized anxiety disorder. Responses are given on a three-point scale (never or almost never, sometimes, often). The total score ranges between 0 and 138, with higher scores indicating greater anxiety severity. The Dutch version of the SCARED has demonstrated good internal consistency (Muris et al., 2007) as well as discriminant validity (Monga et al., 2000), and it remains a commonly used instrument in anxiety research (Sourander et al., 2025).

School-related fear was measured using the School Fear Thermometer (SFT; Heyne and Rollings, 2002), a visual analog scale ranging from 0 (no fear) to 100 (maximum fear). Adolescents rated their fear regarding school attendance for: (i) the next school day; and (ii) their worst day in the past 2 weeks. Researchers have reported high reliability and acceptable validity for the fear thermometer and its variants (Kleinknecht and Bernstein, 1988), and the SFT has demonstrated good test–retest reliability (Heyne, 1999).

Depressive symptoms were assessed using youth and parent versions of the Children’s Depression Inventory-2 [CDI-2; Kovacs, 2016; Dutch version by Bodden et al. (2016)] which continues to be widely used in research (Stewart et al., 2025). The youth version consists of 28 items, each comprising three response statements (0 = ‘not at all’ to 2 = ‘always’). The total score ranges between 0 and 56, with higher scores indicating greater depressive symptoms. The internal consistency of the CDI-2 is high for both the general Dutch population and clinical samples (Bodden et al., 2016). The parent version consists of 17 statements rated on a 4-point scale (0 = ‘not at all’ to 3 = ‘always’), with similar reliability metrics (Kovacs, 2016). The internal consistency of the parent CDI-2 is good, for both the general Dutch population and the clinical population (Kovacs, 2016).

#### 2.3.3 Self-efficacy

Adolescents and parents reported on their own self-efficacy, via the Self-Efficacy Questionnaire for School Situations (SEQ-SS) and the Self-Efficacy Questionnaire for Responding to School Attendance Problems (SEQ-RSAP), respectively. The 23-item SEQ-SS(Heyne et al., 2007) used in this study is a Dutch adaptation of the original 12-item SEQ-SS (Heyne et al., 1998). It measures the strength of the adolescent’s belief that they could cope with potential stressful or anxiety-provoking situations associated with school attendance (e.g., how certain are you that you could go to school if you feel anxious or scared). Items are responded to on a 5-point scale (1 = ‘really sure I could not’ to 5 = ‘really sure I could’). The total score ranges between 23 and 115, and higher scores represent a higher level of self-efficacy. The Dutch SEQ-SS has good internal consistency and convergent validity (Duizer, 2007; Van der Leden, 2008), and translations of the instrument continue to be employed in studies of school attendance challenges (Gonzálvez et al., 2021; Johnsen et al., 2024).

The 25-item SEQ-RSAP (Heyne et al., 2007) assesses the extent of parents’ confidence in their capacity to assist a child facing school attendance challenges, remain composed in such situations, and utilize problem-solving and communication skills to effectively address attendance issues. Items are responded to on a 4-point scale (1 = ‘totally disagree’ to 4 = ‘totally agree), yielding a total score between 25 and 100, with higher scores representing higher levels of self-efficacy. The instrument shows promising convergent validity and good temporal stability (Lavooi, 2010).

### 2.4 Statistical analyses

Data from the completer sample were used to evaluate intervention effects. Repeated measures ANOVAs were conducted for school attendance, anxiety (MASC/MASC-P, SCARED), depression (CDI-2), school-related fear (SFT), and self-efficacy (SEQ-SS, SEQ-RSAP). Greenhouse–Geisser corrections were applied when sphericity assumptions were violated. Pairwise comparisons with Bonferroni correction examined differences between assessment points. Within-subject effect sizes (Cohen’s d) were calculated and interpreted based on Cohen’s (1988) criteria: 0.20 = small effect, 0.50 = medium effect, and 0.80 = large effect. Correlations between self-efficacy and school attendance changes were explored. Missing data were not included in the analyses. Statistical tests were performed using SPSS. All father-report data were excluded from the analyses due to low response rates. As a result, all parent-report measures reflect responses provided by mothers only.

## 3 Results

### 3.1 Effectiveness of the @School intervention

The main outcomes are summarized here to provide an overview of the intervention’s effects, with detailed results presented below. Repeated measures analyses of variance (ANOVA) with pairwise comparisons revealed significant improvements in school attendance, self-reported anxiety (MASC & SCARED), parent-reported anxiety (MASC-P), parent-reported depressive symptoms (CDI-2), and school-related fear (SFT). No significant changes were observed for self-reported depressive symptoms (CDI-2) and self-efficacy (SEQ-SS and SEQ RSAP) (see Table 1). Pairwise comparisons were used to examine specific differences between time points, following the approach used in previous research. The average effect size of improvements between pre-intervention and follow-up (*M Cohen’s d* = 0.82) was larger than the effect size between pre- and post-intervention (*M Cohen’s d* = 0.69), indicating a sustained impact over time.

**Table 1:** Mean scores (standard deviations) of outcome measures at pre-intervention, post-intervention and follow-up.

Variable	Pre-intervention	Post-intervention	Follow-up	*F*	d Post	d FUP
Attendance (%)^a^	17.22 (28.52)	74.85 (30.76)	76.56 (28.26)	*F*(1.45, 18) = 28.17***	1.90	1.97
Self-report						
Anxiety (MASC)^b^	57.17 (19.68)	46.59 (18.63)	41.21 (22.82)	*F*(1.86, 12) = 7.81**	−0.62	−0.62
Anxiety (SCARED)^c^	50.58 (21.37)	41.47 (20.96)	37.00 (25.63)	*F*(2, 12) = 3.41*	−0.51	−0.57
School-related fear (FSSC-R-SI)^d^	54.00 (41.20)	54.00 (41.20)	54.00 (41.20)	*F*(1.23, 8) = 8.59*	−0.83	−0.83
Depression (CDI-2)^e^	16.95 (7.49)	19.24 (18.72)	15.14 (17.79)	*F*(2, 12) = 0.13	0.20	−0.10
Self-efficacy (SEQ-SS-NL)^f^	80.83 (11.48)	90.41 (13.37)	87.14 (26.42)	*F*(1.23, 12) = 1.04	0.61	0.21
Parent-report						
Anxiety (MASC-P)^g^	62.65 (16.85)	51.62 (16.02)	48.36 (18.51)	*F*(2, 12) = 15.11***	−1.00	−1.58
Depression (CDI-2)^h^	16.95 (7.49)	19.24 (18.72)	15.14 (17.79)	*F*(2, 12) = 0.13*	0.20	−0.10
Self-efficacy (SEQ-RSAP)^i^	39.24 (3.63)	41.75 (3.66)	42.62 (4.39)	n.s.	0.66	0.72

Alle parent-report data was based on mothers’ reports given low completion rates by fathers. *p < 0.05; **p < 0.01; ***p < 0.001.

a Percentage School Attendance.

b MASC: Multidimensional Anxiety Scale for Children.

c SCARED: Screen for Children Anxiety Related Emotional Disorders.

d FSSC-R-SI:School-related fear.

e CDI: Children’s Depression Inventory.

f SEQ-SS-NL: Self-Efficacy Questionnaire for School Situations—Dutch Version.

g MASC-P: Multidimensional Anxiety Scale for Children—Parent Version.

h CDI-2: Children’s Depression Inventory-Parent Version.

i SEQ-RSAP: Self-Efficacy Questionnaire for Responding to School Attendance Problems.

#### 3.1.1 School attendance

Repeated measures ANOVA indicated a significant main effect of time on school attendance [*F*(1.45, 18) = 28.17, *p* < 0.001], demonstrating a general increase over time. Pairwise comparisons revealed significant improvements from pre-intervention (*M* = 17.22, *SD* = 28.52) to post-intervention (*M* = 74.85, *SD* = 30.76), but no significant difference between post-intervention and follow-up assessment, indicating that school attendance remained stable after the intervention.

Based on the attendance classification described in the Methods section, at post-intervention, 31.6% of participants met the criteria for good attendance (Tier 1), 5.3% were in Tier 2, and 63.2% had attendance levels categorized as Tier 3. By follow-up, almost half (47.4%) of participants had attendance levels corresponding to Tier 1, 5.3% remained in Tier 2, and 47.4% still had attendance in Tier 3. Further examination of attendance patterns within Tier 3 at follow-up revealed that 37.8% had attendance rates between 40 and 90%, with most in the 50–90% range, indicating varying levels of improvement. Specifically, 10.5% had attendance between 80 and 90, 5.3% between 70 and 80, 5.3% between 60 and 70, and 16.7% between 50 and 60%. Only one participant remained at 0% attendance.

#### 3.1.2 Anxiety and school-related fear

##### 3.1.2.1 MASC, SCARED and MASC-P

Adolescents’ self-reported anxiety (MASC) changed significantly over time [*F*(1.86, 12) = 7.81, *p* = 0.003]. Pairwise comparisons revealed significant reductions from pre-intervention (*M* = 57.17, *SD* = 19.68) to follow-up (*M* = 41.21, *SD* = 22.82 *p* = 0.013), while no significant change was observed between pre- and post-intervention. The second measure of self-reported anxiety (SCARED) also revealed a borderline significant change over time [*F*(2, 12) = 3.41, *p* = 0.050]. Pairwise comparisons indicated that pre-intervention fear scores (*M* = 50.58, *SD* = 21.37) were significantly higher than post-intervention (*M* = 41.47, *SD* = 20.96, *p* = 0.024), but the further reduction from post-intervention to follow-up was not statistically significant.

Parent reports of youth anxiety (MASC-P) also changed significantly over time [*F*(2, 12) = 15.11, *p* < 0.001]. Pairwise comparisons showed that anxiety scores were significantly higher at pre-intervention (*M* = 62.65, *SD* = 16.85) compared to post-intervention (*M* = 51.62, *SD* = 16.02 *p* = 0.017) and follow-up (*M* = 48.36, *SD* = 18.51 *p* < 0.001).

##### 3.1.2.2 SFT

There was a significant main effect of time on fear about going to school, as measured by the SFT [*F*(1.23, 8) = 8.59, *p* = 0.12]. Pairwise comparisons indicated that pre-intervention levels (*M* = 54, *SD* = 41.2 *p* = 0.026) were significantly higher than post-intervention (*M* = 20.85, *SD* = 34.19), with no further significant change from post-intervention to follow-up.

#### 3.1.3 Depressive symptoms

Adolescents’ self-reported depressive symptoms (CDI-2) showed no significant change over time [*F*(2, 12) = 0.13, *p* = 0.88], with a mean score of 16.95 (*SD* = 7.49) at pre-intervention and 15.14 (*SD* = 17.79) at follow-up. However, parent reports of adolescents’ depressive symptoms revealed significant change over time [*F*(2, 12) = 3.97, *p* = 0.032], with significantly higher scores at pre-intervention (*M* = 23.56 *SD* = 4.99) compared to follow-up (*M* = 17.50, *SD* = 5.03).

#### 3.1.4 Self-efficacy

No significant main effect of time was found for self-efficacy, based on either adolescents’ self-reports using the SEQ [*F*(1.23, 12) = 1.037 *p* = 0.345] or parents’ reports of their own self-efficacy using the SEQ-RSAP [*F*(2, 11) = 2.626, *p* = 0.095]. However, for adolescents, a trend towards significance was observed between pre- and post-intervention (*p* = 0.052).

### 3.2 Post-hoc analyses: social anxiety and school attendance

#### 3.2.1 Changes in social anxiety over time

Post-hoc analyses were conducted to further explore changes in social anxiety symptoms over time and their association with school attendance at different time points. Clinically relevant levels of social anxiety were determined using validated cut-off scores on specific subscales. The Social Anxiety subscale of the MASC and MASC-P, and the Social Phobia subscale of the SCARED were used to categorize adolescents’ social anxiety as either clinically elevated or non-clinical. Fisher’s exact tests were conducted to examine changes in social anxiety symptoms over time using validated self- and parent-report measures. At pre-intervention, 33% (*N* = 18) of adolescents scored in the clinical range for self-reported social anxiety as measured by the MASC and 63% (*N* = 19) as measured by the SCARED. By post-intervention, these percentages declined to 18% (*N* = 17) and 42% (*N* = 17), respectively. At follow-up, they further decreased to 14% (*N* = 14) and 21% (*N* = 14). Fisher’s exact tests indicated a significant reduction in social anxiety symptoms from pre- to post-intervention for the MASC (*p* = 0.018) and the SCARED (*p* = 0.009), but no longer statistically significant from pre-intervention to follow-up. Effect size calculations (*Cohen’s d*) showed small-to-moderate differences. For self-reported social anxiety (MASC), *Cohen’s d* was −0.19 at pre-intervention and −0.57 at follow-up. For social phobia (SCARED), effect sizes were −0.25 at pre-intervention and −0.08 at follow-up. Parent-reported social anxiety (MASC-P) showed medium effect sizes at pre-intervention (*d* = 0.45) and follow-up (*d* = 0.63).

Parent-reported adolescent social anxiety decreased from 53% (*N* = 17) in the clinically elevated range at pre-intervention to 19% (*N* = 16) at post-intervention, followed by a slight increase to 21% (*N* = 14) at follow-up. None of these changes were statistically significant.

#### 3.2.2 Association between social anxiety and school attendance

Independent samples t-tests were conducted to compare school attendance rates between adolescents with and without clinically elevated social anxiety. At pre-intervention, adolescents with clinically elevated social anxiety on the MASC had a mean school attendance of 22.03% (*n* = 6, *SD* = 36.57) compared to 16.25% (n = 12, SD = 26.12) for those without [*t*(16) = −0.388, *p* = 0.703]. At follow-up, adolescents with clinically elevated social anxiety had an attendance rate of 91.18% (*n* = 2, *SD* = 5.81) compared to 74.78% (*n* = 12, *SD* = 30.14) for those without [*t*(12) = −0.743, *p* = 0.472].

## 4 Discussion

This effectiveness study evaluated outcomes following the delivery of the @School intervention in a real-world setting, focusing on neurodiverse adolescents experiencing ER-SAC. While previous research has focused on predictors of school non-attendance among this group (Adams et al., 2025), less is known about which interventions benefit these adolescents. Our findings help close this knowledge gap by providing evidence on a school-based CBT approach tailored to support neurodiverse adolescents experiencing ER-SAC.

The study built upon the Heyne et al. (2011) efficacy study, which was conducted under ideal research conditions and did not include neurodiverse adolescents. Key findings from the current study suggest that the @School intervention—a modular, developmentally-sensitive CBT integrating adolescent-focused intervention, structured parent involvement, and collaboration with school staff—was associated with significant positive outcomes that were maintained at five-month follow-up when delivered in a real-world educational setting. These outcomes included increased school attendance, reduced anxiety symptoms and school-related fear, and reduced depressive symptoms as reported by parents. The following discussion considers these and related results.

### 4.1 Interpreting the increase in school attendance

School attendance improved substantially following the @School intervention. Average attendance rose from 17% at pre-intervention to 80% at follow-up. Only one adolescent (5%) had 0% attendance at follow-up. In comparison, the Heyne et al. (2011) efficacy study reported an increase from 15% at pre-intervention to 48% at follow-up, with 45% of adolescents remaining entirely absent. These outcomes suggest that delivering the intervention within a school setting may offer added benefits for improving attendance.

One factor that may explain the apparent higher attendance rates in the present study compared to Heyne et al. (2011) is the structured, school-based delivery of the intervention, which facilitated sustained support and engagement from school staff. While school involvement was also present in the 2011 study, the current approach emphasized direct collaboration between therapists and school staff as a core element of the intervention, ensuring real-time adaptations and ongoing coordination between the two professional groups. This reflects a shift from a more peripheral role for schools in the Heyne et al. (2011) study, to a central, proactive, and integrated role in the current study—potentially contributing to sustained attendance improvements at follow-up. The integration of individual, family, and school-based support in the @School intervention aligns with the KiTeS model (Melvin et al., 2019), which offers a bioecological framework for understanding school attendance difficulties. This model emphasizes that attendance problems emerge from the dynamic interplay between individual vulnerabilities and contextual factors across multiple ecological systems, including family, school, and the broader environment. This ecological perspective helps explain why addressing support needs, across the various ecological systems, may have been particularly effective for neurodiverse adolescents facing complex, layered attendance challenges.

The centralized structure of the intervention, in which healthcare and education providers worked together in a single integrated organization, was seen by both parents and adolescents as helpful for streamlining communication and coordination. This integrated model facilitated real-time collaboration between school staff and mental health professionals, ensuring adolescents received cohesive and timely support. While models in which educational and mental health teams operate in separate organizations can also be effective, integration within a single organization allowed for smoother information exchange through regular in-person interaction and shared access to adolescent monitoring systems. Also, school-based therapists were well-informed about the curriculum, the constraints teachers face, and the educational goals set for the adolescents. In turn, educational staff had, while respecting confidentiality through explicit consent procedures, access to insights from the therapeutic process regarding the challenges faced by adolescents and their families, enabling more tailored academic and emotional support. The shared organizational structure fostered consistency in how the intervention was delivered by psychologists and teaching staff, it supported alignment between educational and therapeutic goals, and reduced the coordination challenges often encountered when services span multiple agencies.

The Tiered system for addressing attendance and absence offers a broader lens for interpreting the current results (Kearney, 2016; Kearney and Graczyk, 2020). Prior to the intervention, all adolescents (100%) met the criteria for Tier 3 ‘chronic absence’ (i.e., ≤ 0% attendance). At post-intervention, the attendance of 32% of adolescents met the threshold for Tier 1 ‘good attendance’ (i.e., >95%), increasing to 47% at follow-up. Viewed this way, the intervention had a lasting positive effect for nearly half the participants, although the attendance rates of 47% remained in Tier 3 with ‘chronic absence’ at follow-up, indicating ongoing attendance challenges for a subset of adolescents.

A closer examination revealed that 38% of the adolescents with chronic absence had attendance rates between 40–90%, indicating that some adolescents made substantial progress despite not attending school at a level warranting Tier 1 or 2 classification. The current outcomes align with prior research showing that interventions for school attendance can yield substantial improvements but may not be equally effective for all adolescents (Finning et al., 2019; Heyne, 2023; Maynard et al., 2018).

Notably, the tiered classification system referred to above is based on relatively conservative cut-off scores. As emphasized by Melvin et al. (2025), there is currently no international consensus on the thresholds used to define chronic absence, and in some countries, adolescents with 80% attendance would be considered to have acceptable or even good attendance (Kreitz-Sandberg et al., 2022) These contextual differences may influence the interpretation and perceived success of interventions across countries (Heyne et al., 2022a), highlighting the importance of cultural and policy contexts when interpreting school attendance outcomes.

Even with substantial progress for a notable proportion of the adolescents in the current study, full attendance remained out of reach for some —highlighting the need to consider individual and contextual factors when interpreting outcomes. That some adolescents made notable progress in building their school attendance but still remained within tier 3 suggests that long-term attendance outcomes may be influenced by factors beyond the intervention itself, such as mental health, family support, and school climate (Finning et al., 2019; Kearney, 2016). Additionally, improvement in school attendance may take longer for some adolescents. Clinicians involved in this study observed that some adolescents were able to increase their school attendance but reached a threshold where further in-person attendance was not feasible due to factors such as sensory sensitivities or emotional exhaustion. In such cases, adjustments such as part-time attendance combined with structured home-based learning were necessary to sustain learning. These adaptations enabled adolescents to regulate sensory input while maintaining academic progress, and they highlight the importance of flexible attendance strategies within school-based interventions for neurodiverse adolescents (Adams et al., 2025; White et al., 2024).

### 4.2 Interpreting the reduction in emotional distress

#### 4.2.1 Reduced anxiety

Reductions in anxiety symptoms, as measured by the SCARED and MASC, suggest that the intervention was effective not only in improving school attendance but also in alleviating emotional distress, which is a defining feature of ER-SAC. Adolescents reported substantial decreases in anxiety and school-related fear, with effect sizes ranging from medium to large. Parent-reported outcomes (MASC-P) reflected similar reductions in the adolescents’ anxiety, indicating both subjective and observed improvement emotionally. The current findings align with those from (Heyne et al., 2011), which also reported reductions in anxiety and school-related fear among participants. This consistency suggests that the @School intervention may be effective in alleviating both anxiety and school-related anxiety. These findings align with prior research showing that school-based interventions incorporating structured exposure can effectively reduce anxiety-driven avoidance behaviors (Perihan et al., 2021).

#### 4.2.2 Disparate reports of reduced depression

The effects on depressive symptoms were not consistent across informants. While self-reported depressive symptoms did not show significant change over time, parent-reported symptoms decreased significantly from pre-intervention to follow-up, with a large effect size. Notably, the pattern of findings in the present study differs from those of Heyne et al. (2011), who reported reductions in depressive symptoms across adolescent and parent reports. The following discussion considers the lack of change in self-reported depression and the substantial parent-reported improvements.

First, the wide variability in self-reported depressive symptoms indicates that while some adolescents improved, others did not, which likely obscured group-level trends.

Second, two factors may affect the validity of self-reported depressive symptoms in neurodiverse adolescents: symptomatic overlap and challenges in emotional introspection. Although the CDI-2 is a widely validated tool for assessing depressive symptoms in adolescents, research suggests that it may not fully capture the unique manifestations of depression in neurodiverse adolescents (Cassidy et al., 2018). Some behaviors traditionally associated with depression, such as social withdrawal, disruptions in sleep–wake cycles, and reduced activity levels, may overlap with neurodiverse characteristics rather than reflecting a clear depressive state. For example, disrupted sleep patterns may not necessarily indicate depression but instead could result from sensory processing sensitivities common among neurodiverse adolescents. This symptomatic overlap complicates the interpretation of changes in depressive symptomatology. In the current study, the CDI-2 may have captured neurodiversity-related behaviors rather than mood-related distress, thereby masking meaningful improvements. Furthermore, standard self-report tools rely on an individual’s ability to reflect on and report internal emotional states. Pezzimenti et al. (2019) note that neurodiverse adolescents may experience difficulties with emotional introspection. Consequently, stable CDI-2 scores may reflect limitations in emotional self-awareness rather than a lack of change in psychological functioning.

Third, it is possible that mood-related difficulties require a longer timeframe to decrease compared to anxiety symptoms. Research suggests that mood-related difficulties in neurodiverse adolescents may persist longer than anxiety symptoms due to their interaction with cognitive rigidity, social stressors, and emotional regulation challenges (Pezzimenti et al., 2019). Beyond school stressors, factors such as sensory sensitivities, difficulties in forming social connections, and struggles with emotional regulation further contribute to these persistent mood disturbances (Perihan et al., 2021). Given that depression in ASD is often tied to challenges in processing social information and heightened stress sensitivity, adjustments were made throughout the implementation of the intervention to meet the specific needs of neurodiverse adolescents. These adaptations are now being specified in the @School protocol to support future application.

Parent reports provided a valuable complementary perspective, revealing significant reductions in depressive symptoms not captured in adolescent self-reports. Parents reported higher levels of depressive symptoms than adolescents at pre-intervention (*M* = 23.56, *SD* = 4.99), with scores significantly decreasing by follow-up (*M* = 17.50, *SD* = 5.03). However, follow-up scores remained in the clinical range, suggesting that while improvement was observed, on average, depressive symptoms had not fully resolved.

Understanding the discrepancy between parent and adolescent reports requires consideration of how each informant assesses and interprets depressive symptoms. Parents typically evaluate their child’s emotional well-being based on observable behaviors, such as engagement in social activities or sleep patterns, whereas adolescents assess their internal emotional state (Olino et al., 2018). These behaviors may not necessarily indicate symptoms of a depressive disorder but could reflect the consequences of prolonged school absence, including reduced peer interactions, decreased daily structure, and altered bedtimes. As school attendance improves, these behaviors often normalize, leading parents to perceive emotional improvement even when internal distress persists (Smith and White, 2020). This aligns with our findings, where parent reports indicated significant reductions in depressive symptoms, while self-reports showed more stability.

These findings highlight the complexity of assessing depressive symptom changes in neurodiverse adolescents, where discrepancies between parent and adolescent reports reflect different but equally valuable perspectives on emotional well-being. Rather than being seen as contradictory, these perspectives offer a more comprehensive understanding of the intervention’s impact.

### 4.3 Interpreting the lack of change in self-efficacy

The improvements in school attendance and emotional wellbeing were not accompanied by improvements in adolescents’ or parents’ self-efficacy, with scores remaining stable across all time points. This contrasts with the findings of Heyne et al. (2011), who reported significant increases in both adolescent and parent self-efficacy following the intervention. However, we noted a trend towards increased adolescent self-efficacy between pre- and post-intervention (*p* = 0.052), suggesting that some individuals may have experienced initial gains. Several factors may help explain why self-efficacy did not significantly improve, including challenges related to introspection in neurodiverse adolescents, and the role of external support in shaping self-efficacy beliefs.

The measurement of self-efficacy in neurodiverse adolescents presents specific challenges. Weiss et al. (2015) found that individuals with ASD often struggle with introspective assessments of their own abilities, which could result in difficulties accurately reporting changes in self-efficacy. Moreover, Silva et al. (2023) emphasize that self-efficacy in neurodiverse individuals is not solely influenced by performance but also by sensory sensitivities, executive functioning difficulties, and social challenges. The SEQ-SS was not developed with these issues in mind and may therefore fail to adequately account for self-efficacy among neurodiverse young people displaying ER-SAC.

The lack of significant change in parental self-efficacy aligns with prior research suggesting that parents of adolescents with ASD often experience lower self-efficacy due to ongoing stress and the complexity of managing their child’s needs, which can persist despite interventions (Weiss et al., 2015).

Finally, research suggests that self-efficacy develops when individuals attribute their successes to their own efforts rather than external support (Usher and Pajares, 2008). The structured nature of the intervention may have provided adolescents with external reinforcement but limited opportunities for independent problem-solving, which is crucial for fostering self-efficacy. Silva et al. (2023) highlight that in neurodiverse populations, high levels of external support may inadvertently reduce an individual’s sense of autonomy and personal agency, potentially explaining the lack of significant self-efficacy gains.

### 4.4 Interpreting the role of social anxiety in school attendance outcomes

In interpreting our study’s findings, we explored whether social anxiety symptoms influenced school attendance outcomes among participating adolescents. Social anxiety can create significant barriers to full participation in school life, and studies show links with absence from school (Finning et al., 2019; Gonzálvez et al., 2019; Tekin and Aydın, 2022). In the efficacy study by Heyne et al. (2011), 65% of adolescents had a social anxiety diagnosis at pre-intervention; 40% with a primary diagnosis and 25% with a secondary diagnosis. Of those with a social anxiety diagnosis at pre-intervention, 54% still met criteria at follow-up. Moreover, school attendance at follow-up was significantly lower among those with social anxiety disorder at follow-up (18% attendance) compared to those without this diagnosis at follow-up (68%).

Unlike that study, which relied on clinical diagnoses, we assessed symptoms of social anxiety or social phobia (hereafter ‘social anxiety symptoms’) using validated self- and parent-report measures (MASC, MASC-P, and SCARED). Adolescents were classified as having either clinically elevated or non-clinical levels of social anxiety based on established cut-off scores. No significant differences in school attendance were found between adolescents with and without clinically elevated symptoms at pre-intervention, post-intervention, or follow-up. This might suggest that, in contrast to Heyne et al. (2011), social anxiety symptoms did not act as a barrier to school attendance in the current study.

Although both the current study and the 2011 study evaluated the @School intervention, important differences in how the intervention was delivered may help explain the differing outcomes. The school-based approach in the current study on the @School intervention likely played a pivotal role in mitigating the impact of social anxiety by providing several advantages. It is worth noting that in the current study several adolescents transitioned into a specialized education setting where accommodations for neurodiversity were provided. These tailored supports may have reduced environmental stressors, allowing adolescents to maintain school participation regardless of social anxiety levels. Adolescents who transitioned to specialized education settings often experienced a sense of normalization, recognizing they were no longer an exception compared to their peers, potentially further reducing social anxiety. Furthermore, the intervention as employed in the current study facilitated close coordination between mental health professionals and educational staff, ensuring precise tailoring of exposure tasks matched to each adolescent’s readiness level. Teachers were thus able to provide a secure and predictable environment, helping adolescents feel safer during these steps.

While the school-based intervention may have reduced the impact of social anxiety symptoms on attendance, another factor that could explain these findings is the way social anxiety was defined and measured in this study. Heyne et al. (2011) focused on adolescents with a formal clinical diagnosis of social anxiety disorder, while the present study assessed social anxiety symptom severity using self- and parent-report measures. Adolescents with a clinical diagnosis may experience more severe and persistent anxiety-related school avoidance, making their school attendance patterns more distinct from those without social anxiety disorder.

### 4.5 Contextualizing outcomes through comparative perspectives

To contextualize our findings beyond the preceding comparisons with the 2011 efficacy study, a recent evaluation of the Back2School program (B2S) (Daniel B Johnsen et al., 2024) provides a useful point of reference. B2S, also a modular CBT intervention, draws on components from the @School intervention (Heyne and Sauter 2013; Heyne et al.,2014) and the When Children Refuse School protocol (Kearney and Albano, 2018). Whereas @School was designed specifically to address ER-SAC, B2S was developed to address a broader range of SACs and was described as applicable for “truancy, school refusal, school withdrawal, and school exclusion” (Johnsen et al., 2024).

Notably, B2S was associated with improvements in adolescent self-efficacy, whereas this was not observed in our study (see Section 4.3). On the other hand, average school attendance at three-month follow-up in the B2S study was 58% (measured in school hours), compared to 80% at 5.4 months follow-up intervention in the current study (also measured in school hours).

These differences may reflect variation in numerous factors, including the focus of the intervention and the level of collaboration with schools. Regarding focus, @School was specifically designed for adolescents experiencing ER-SAC, whereas B2S was developed to address absence more generally. Regarding school involvement, @School was fully embedded within an educational setting, with mental health and education professionals working in tandem. B2S involved more limited collaboration, consisting of an average of three 1-hour meetings between the mental health professionals delivering B2S and school personnel.

While comparisons across studies must be approached with caution, given differences such as in population (e.g., country, age) and timing of follow-up, they can nevertheless inform reflection on how intervention focus and setting may shape outcomes. For researchers, these kinds of cross-study comparisons can guide future investigations into which components drive specific outcomes, for whom, and under what conditions. For practitioners and program developers, they underscore the importance of selecting or adapting interventions to match both the needs of the target population and the intended outcomes, and of carefully considering the nature and extent of collaboration between education and mental health sectors to optimize effectiveness.

### 4.6 Strengths and limitations

This study offers several strengths. First, the intervention targeted the adolescent directly through individual support, while also addressing the family and school systems, ensuring that support strategies address not only the required intensity of intervention, but also the specific nature of the attendance problem being targeted. Second, it focused on neurodiverse adolescents, a group at heightened risk for school non-attendance but often underrepresented in intervention research. Third, delivering the intervention in a school-based setting bridged the gap between mental health care and education, enhancing accessibility and real-world relevance. This contextualized implementation, with active involvement of educational staff, likely contributed to sustained improvements at follow-up by aligning academic accommodations with mental health needs.

Despite these strengths, the study has several limitations. First, the small sample size limits statistical power, reducing confidence in detecting smaller effects. It also restricts generalizability, as the findings may not extend to broader populations. Second, the study lacks a control group, preventing definitive conclusions about the intervention’s causal effects. Third, the absence of longer-term follow-up data restricts our ability to assess the durability of improvements in school attendance and emotional well-being beyond 5 months post-intervention. Lastly, the intervention was delivered in a specialized setting by psychologists with extensive clinical experience. While this likely contributed to consistent implementation and may have supported treatment outcomes, it also raises questions about replicability in less specialized or less experienced contexts.

### 4.7 Implications for research and practice

The findings of this study carry important implications for both research and practice. Future research should explore strategies to enhance self-efficacy within ER-SAC interventions, particularly for neurodiverse adolescents. Targeted approaches, such as structured mastery experiences, problem-solving exercises, and coping skills training, may help build confidence in managing school-related challenges (Usher and Pajares, 2008). Including school staff as informants could also enrich outcome evaluation by capturing observations of adolescent support needs and progress. Moreover, qualitative methods, such as interviews with adolescents, parents, and educators, could offer deeper insight into individual experiences of intervention and guide refinement of intervention strategies.

The Multi-Dimensional Multi-Tiered System of Supports (MD-MTSS) framework offers a helpful conceptual foundation for applying the current findings in school contexts. This framework conceptualizes attendance support as a three-dimensional model that combines levels of intervention intensity (Tiers) with types of attendance problems. While the field has traditionally focused on levels of support, our findings underscore the importance of also considering the nature of the attendance challenge. Attending to both the severity and nature of attendance problems allows for more precise intervention matching, ensuring that support strategies address not only the required intensity of intervention, but also the specific nature of the attendance problem being targeted (Kearney and Graczyk, 2020).

To implement this effectively, schools should promote close collaboration between educators and mental health professionals, fostering alignment between academic expectations and emotional support systems. Training school staff to identify attendance barriers early and respond with appropriate interventions is essential for improving outcomes, particularly for neurodiverse adolescents who often face multifaceted attendance challenges.

## 5 Conclusion

This study provides support for the effectiveness of the @School intervention for neurodiverse adolescents experiencing ER-SAC, when implemented in a school setting. A school-based, modular approach, involving adolescents, parents, and school staff contributed to improvements in school attendance and meaningful gains in emotional well-being. Differences between parent- and adolescent reported depressive symptoms, along with the absence of significant gains in self-efficacy, highlight the challenges of accurately assessing internal change processes in neurodiverse adolescents, particularly emotional distress and self-beliefs. These findings underline the need of more sensitive tools that can better capture such changes and inform more targeted support within interventions for neurodiverse adolescents experiencing ER-SAC. In sum, a structured and integrated approach, such as @School, offers a promising model for addressing both the emotional and the academic needs of neurodiverse adolescents experiencing emotion-related school attendance challenges, particularly when implemented within a school setting.

## Data Availability

The data are not publicly available due to ethical and legal restrictions. Participants did not provide consent for their data to be shared outside the research team. For questions regarding the dataset, please contact the corresponding author: Evelyne Karel, Evelyne.karel@berkenschutse.nl.
