# Supplementary material for: School-based support for emotion-related attendance challenges: effectiveness of @School when implemented with neurodiverse adolescents, their parents, and school staff
Source: Front Psychol. 2025 Jun 23;16:1613712. doi: 10.3389/fpsyg.2025.1613712 (PMC12230860; doi:10.3389/fpsyg.2025.1613712)
Supplement: Supplementary file 1 [file Supplementary_file_1.docx]

**Supplementary Material 1: Structure and Content of the @School Intervention**

Introduction

The @School intervention is a developmentally sensitive, modular cognitive-behavioral therapy (CBT) program designed to support adolescents experiencing Emotion-Related School Attendance Challenges (ER-SAC), while also providing support for parents and school staff. Developed by Heyne et al. (2013), the intervention addresses emotional, cognitive, behavioral, familial, and systemic factors contributing to ER-SAC. Its modular structure allows for individual tailoring based on a shared case formulation developed collaboratively with the adolescent, parents, and school staff.

The intervention comprises youth-focused modules, parent-focused modules, and school-related strategies, each aimed at enhancing emotional regulation, promoting graduated school re-engagement, empowering families, and facilitating academic and social adjustment and well-being.

This document provides a description of the modules within the @School intervention, as originally developed. For further information about implementing the intervention—including guidance on assessment, intervention planning, module selection and pacing, and working with young people, parents, and schools—see Heyne et al. (2013).

Adapted in part from: Heyne, D. A., & Sauter, F. M. (2013). School Refusal. In C. A. Essau & T. H. Ollendick (Eds.), The Wiley-Blackwell Handbook of the Treatment of Childhood and Adolescent Anxiety (pp. 471–517). John Wiley & Sons, Ltd.

**Youth Modules**

*Y1. Reviewing the Overall Plan*

This initial module focuses on establishing a strong collaborative relationship between the adolescent and the therapist. The therapist shares the personalized case formulation, helping the adolescent understand the factors contributing to their school attendance challenges. Through this transparent dialogue, motivation for engagement in therapy is fostered, and the adolescent is positioned as an active participant in their change process.

*Y2. Putting Problems in Perspective*

Adolescents receive psychoeducation about emotional functioning, anxiety, depression, and the cognitive-behavioral model. They learn how emotions, thoughts, and behaviors interact, and how these patterns can contribute to school attendance difficulties. Normalizing their experiences reduces shame and builds readiness for intervention.

*Y3. Thinking about the Teenage Years (Optional)*

This optional module explores developmental tasks of adolescence, including striving for autonomy, forming an identity, and managing peer relationships. Adolescents reflect on how these typical challenges may intersect with their school experiences, supporting self-understanding and developmental growth.

*Y4. Setting Goals*

Adolescents collaborate with their therapist to identify meaningful and realistic goals for therapy. Goals are framed positively, linked to the adolescent’s values, and provide a motivational roadmap for the intervention.

*Y5. Solving Problems*

This module introduces a structured problem-solving approach: identifying problems clearly, brainstorming solutions, weighing pros and cons, and planning action steps. Skill development in this area enhances coping with both school-specific and broader life challenges.

*Y6. Managing Stress*

Adolescents learn stress management techniques such as diaphragmatic breathing, progressive muscle relaxation, and mindfulness strategies. Early recognition of stress symptoms and proactive coping are emphasized.

*Y7. Dealing with Cognition*

This cognitive restructuring module helps adolescents to identify and challenge unhelpful automatic thoughts, replacing them with more balanced and realistic alternatives. This supports emotional regulation and behavioral flexibility. The implementation of this module is guided not only by the relevance of cognitive factors in the case formulation, but also by the adolescent’s developmental stage and readiness for cognitive work. Developmentally sensitive adjustments may be required in how cognitive concepts are introduced, practiced, and applied.

*Y8. Dealing with Social Situations (Optional)*

Adolescents struggling with social anxiety or those facing broader challenges in social interactions, receive targeted support in developing social competence, assertiveness, and effective peer interaction skills. Behavioral experiments and graded exposure tasks are used to build confidence.

*Y9. Dealing with Depression (Optional)*

Adolescents presenting with depressive symptoms engage in behavioral activation strategies, aimed at increasing positive reinforcement through structured activities. Thought-challenging techniques are also employed to address cognitive distortions.

*Y10. Solving Family Problems (Optional, Joint Session)*

In joint sessions with parents, adolescents learn and practice structured communication and collaborative problem-solving skills. The focus is on addressing family dynamics that may hinder school attendance or emotional well-being.

*Y11. Attending School*

This core module centers on the gradual return to school using exposure-based principles. A stepwise attendance plan is developed, integrating coping skills, support strategies, and positive reinforcement mechanisms.

*Y12. Promoting Progress*

As treatment concludes, adolescents reflect on their achievements, identify ongoing coping strategies, and formulate relapse prevention plans. Future challenges are anticipated, and proactive responses are rehearsed.

**Parent Modules**

*P1. Reviewing the Overall Plan*

This module aims to actively involve parents from the start by presenting the case formulation and treatment plan, developed collaboratively with the adolescent, parents and school staff. Parents are supported in understanding the multifactorial contributors to their child's school attendance problems, including emotional, familial, and systemic influences. The emphasis is on building a sense of partnership between therapists and parents and preparing parents to fulfill an active role in the intervention.

*P2. Putting Problems in Perspective*

Parents are provided with psychoeducation on anxiety, depression, and school refusal behaviors, emphasizing the normalcy of emotional challenges and the utility of CBT principles. This fosters empathy toward their child’s difficulties and reduces critical or punitive reactions. Parents also learn how their own responses can either reinforce or help reduce school avoidance.

*P3. Thinking about the Teenage Years (Optional)*

This optional module educates parents about normative adolescent developmental tasks—such as striving for autonomy and forming identity—and how these intersect with school attendance challenges. Parents explore ways to support autonomy while maintaining necessary boundaries, thereby reducing conflict and promoting developmental growth.

*P4. Setting Goals*

Parents collaborate with therapists to set clear, meaningful goals for supporting their child’s school re-engagement. Goals are aligned with the adolescent's individual plan and include specific actions parents can take to reinforce attendance and emotional regulation efforts at home.

*P5. Reducing Maintenance Factors*

This module helps parents identify home and family environment factors that may unintentionally maintain school refusal, such as accommodating avoidance behaviors. Strategies to modify these patterns are introduced, empowering parents to create a supportive, change-facilitating environment.

*P6. Giving Effective Instructions*

Parents are supported and guided to deliver instructions in a clear, firm, and supportive manner, minimizing ambiguity and resistance. Skills such as gaining attention before speaking, providing specific and manageable tasks, and using positive reinforcement are emphasized.

*P7. Responding to Behavior*

This module teaches parents to systematically reinforce desired behaviors (e.g., attending school) and reduce reinforcement of avoidance behaviors. Techniques include using reward systems, planned ignoring of minor undesired behaviors, and implementing logical consequences.

*P8. Bolstering a Young Person’s Confidence*

Parents are coached on how to support their child’s gradual re-engagement with anxiety-provoking situations, including school attendance. Emphasis is placed on encouraging efforts, celebrating small successes, and modeling resilience in the face of setbacks.

*P9. Bolstering a Parent’s Confidence (Optional)*

Recognizing that parents may experience anxiety and helplessness themselves, this module focuses on strengthening their coping skills. Parents learn emotional regulation strategies and ways to manage their own stress to maintain consistency and optimism when supporting their child.

*P10. Solving Family Problems (Optional, Joint Session)*

In joint sessions with their child, parents practice structured communication and problem-solving skills. The goal is to collaboratively address family dynamics that may be contributing to the child’s difficulties and to foster healthier interaction patterns.

*P11. Facilitating School Attendance*

This module equips parents with skills to advocate for their child’s needs within the school setting, collaborate with school staff, and monitor progress in school re-engagement efforts. It emphasizes proactive, solution-focused communication with educators.

*P12. Promoting Progress*

In the final parent module, attention turns to sustaining gains after treatment concludes. Parents work with therapists to develop relapse prevention plans, identify potential future challenges, and formulate action plans to maintain positive progress.

**School Collaboration Modules**

*S1. Orientation to the intervention*School staff are guided through the shared case formulation to enhance their understanding of the contributing factors to the young person’s school refusal. The selected CBT modules are reviewed with attention to their relevance for the case. This meeting also clarifies the roles of the adolescent, parents, school staff, and clinicians in the intervention, fostering a coordinated and shared responsibility for change.

*S2. Organizational issues*A concrete attendance plan is developed, often involving part-time attendance as a stepwise approach to re-engagement. A coordinator is identified within the school to liaise with other staff, monitor progress, and maintain communication with the clinician. This structure supports consistency and early identification of setbacks.

*S3. Emotional issues*In collaboration with school staff, strategies are developed to reduce and respond to emotional distress experienced by the adolescent during school hours. Common strategies include ensuring easy access to a trusted staff member, normalizing emotional reactions, and reinforcing coping behavior. These strategies help prevent escalation and support persistence.

*S4. Behavioral issues (Optional)*Where relevant, school staff engage in problem-solving discussions about how to respond to externalizing behavior linked to school refusal. The aim is to avoid punitive measures and instead use constructive responses, such as alternative arrangements or the use of logical consequences.

*S5. Academic issues (Optional)*Staff consider temporary adjustments to academic expectations to accommodate the young person’s needs during re-engagement. This may include reduced workload, flexible deadlines, or deferral of non-core subjects such as physical education. Such accommodations support a smoother academic reintegration.

*S6. Social issues (Optional)*Social difficulties are addressed through joint planning with staff. Interventions may include buddy systems to increase peer support, provision of structured break-time activities, and strategies for preventing or responding to bullying. These supports help the adolescent feel safer and more connected at school.

*S7. Promoting progress*As attendance improves, school staff participate in planning for maintenance and relapse prevention. Meetings focus on sustaining progress, gradually fading supports, and identifying early warning signs of setbacks. Strategies are established to manage potential lapses constructively.

**References:**Heyne, D. A., & Sauter, F. M. (2013). School Refusal. In C. A. Essau & T. H. Ollendick (Eds.), The Wiley-Blackwell Handbook of the Treatment of Childhood and Adolescent Anxiety (pp. 471–517). John Wiley & Sons, Ltd.
